# Helping parents to cope with infant regulatory disorders

**DOI:** 10.3389/frcha.2024.1322962

**Published:** 2024-03-26

**Authors:** Ian St James-Roberts, Clare Llewellyn

**Affiliations:** ^1^ Thomas Coram Research Unit, University College London, London, United Kingdom; ^2^ Research Department of Behavioural Science and Health, University College London, London, United Kingdom

**Keywords:** infant crying, infant sleep, infant feeding, regulatory disorders, parenting

## Abstract

The term Regulatory Disorders (RDs) refers to infants and young children who cry a lot, have poorly organised sleep-waking, or whose feeding is impaired. The characteristic they share is a failure to acquire autonomous self-control of these key behaviours, which most children develop in the first postnatal year. The concept of RDs is helpful in highlighting this question of how infant self-regulation is, or isn't, accomplished, in drawing these characteristics together and distinguishing them from others, and in focusing research and clinical attention on a common, but relatively neglected, set of concerns for families. The main focus for research into RDs has been on the nature and causes of the infant behaviours involved. Here, the aim is to highlight the part played by parents, since that is central to the provision of clinical services for RDs. Three points are made. (1) The contributions of parents include: detection and monitoring of RDs; generating the resulting healthcare service costs; maintaining their own wellbeing, since that is in their infant’s best interest; delivering interventions to help infants and families, which are almost always provided by parents. Parenting may be involved in maintaining RDs in some cases. (2) Substantial differences exist between infant RD cases in the behaviours involved, the age at which they present, the complexity and severity of the RD, and in their persistence over time and age. Most cases have one, rather than all three RDs, making them the most common type seen by clinicians. Evidence is summarised that interventions tailored to fit the RD involved can be effective in these cases. Multiple and sustained RDs are much rarer, but associated with greater risk of long-term psychological and behavioural impairments. They are a priority, but less is known about the contributions of parenting and child factors to these cases. (3) The focus on parents with infants requires joined-up paediatric and adult mental health services. After summarising three main requirements for such services, an example designed to fulfil these requirements is described to illustrate what a service for supporting families with RDs might look like.

## Introduction

One of the most important tasks for young children is to develop control of their own sleep, waking and feeding behaviours. This transition to infant autonomous regulation of these crucial behaviours does not always go smoothly. The term Regulatory Disorders refers to cases involving prolonged infant crying, poorly organised sleep-waking, or impaired feeding. Surveys have found that more than a third of infants exhibit at least one of these behaviours, making them common challenges for parents and families and costly concerns for healthcare systems (1, 2). For brevity, we will refer to infant and child Regulatory Disorders and their synonyms Regulatory Disturbances and Regulatory Problems as RDs. Together with this collection of articles, the publication of three systematic reviews, the most recent including meta-analysis of 30 studies, is evidence of the growing interest in RDs (3–5).

Historically, the main focus for research into RDs has been on the nature and causes of the infant behaviours involved. In contrast, the aims in this article are to: (1) Highlight the importance of parental contributions and the need for equal attention to them; (2) Propose that the focus for primary healthcare services should include supporting parents in gaining coping skills; (3) Summarise the evidence that methods developed by psychologists and psychiatrists to support parental mental health, including cognitive behaviour therapy, provide cost-effective techniques for this purpose.

Because our focus is on parents, it is worth saying at the outset that we do not intend to imply that parents cause infant RDs. There is ample evidence that all three RDs are associated with parental factors, such as anxiety, depression, and parenting behaviours, particularly in clinical samples (6–15). However, these are observational studies, which leave the direction of causation unclear. That is, these studies cannot establish whether the parenting factor was the initial trigger for the infant RD, whether the infant RD caused the parental problem, or whether a third factor (such as genetic confounding) contributed to both parental and infant elements. We cannot assume that all infants are inherently good at regulating their crying, sleep, or feeding. Some may be slower to mature or learn, or have a strong biological basis to their behaviour, so that they need extra parenting support compared to most infants. There is evidence, for example, that some babies cry a lot in spite of highly sensitive and responsive parenting (16, 17).

In principle, randomised controlled trials (RCTs), which intervene to change parenting behaviours in one group, compared to a control group, provide the strongest evidence about causation. We will examine the available RCTs in more detail below, but it is sufficient here to note that the findings are complex and leave causal directions uncertain. Similarly, a recent systematic review of the evidence was unable to conclude whether inadequate parenting contributes to infant RDs (5). Behaviour genetic studies, which provide a powerful tool for examining causation, indicate too that RDs are complex problems with multiple interacting causes (18–21). Lastly, there is evidence that factors within young infants are often involved (22). However, these factors are heterogeneous, that is, they vary greatly between cases. Understanding that diversity is essential for interventions designed to help infants and their families, particularly where parents are responsible for delivering the interventions, as they usually are. We will return to develop these points below.

## Heterogeneity among infant and child RDs

The term and concept of RDs is helpful in identifying characteristics shared by some young children, distinguishing them from other characteristics such as intellectual impairments, and focusing research and clinical attention on a common, but relatively neglected, set of concerns for families. As noted above, the three RD types share a focus on the acquisition of autonomous self-control, that is, young children's ability to regulate their own crying, sleeping and feeding behaviours.

Although the notion of RDs is helpful, there are substantial differences between individuals in the behaviours involved, the age at which they present, the complexity and severity of the RD, and in their persistence over time and age. Infant crying and parental concern about unexplained or “excessive” crying peaks at about 5–6 weeks of age, occurs most often in the afternoon and evening, and reduces substantially by 12 weeks of age (23–26). In contrast, infant sleep problems involve waking at night in older infants (27, 28). Newborn infant sleeping periods are short and vary little between day and night. By three months of age, most infants begin to sleep for longer periods in the night than day, and by six months about 70% of them have the long night-time sleep periods typical of mature sleep-waking and highly valued by parents (27). The approximately 30% of infants who do not make this transition are said to have poorly regulated sleep, or “sleeping problems” (27).

The development of infant feeding self-regulation is particularly complex. From birth, infants need to coordinate sucking, breathing and swallowing to establish effective feeding. Sucking is initially automatic and reflexive, but becomes voluntary by four months of age. Infants must learn to recognise, respond to, and express their internal biological signals of hunger and satiety to caregivers to regulate milk intake. At about six months complementary feeding begins, during which infants acquire oral motor skills in biting, chewing and swallowing a range of textures and flavours, as well as the ability to self-feed. Disorders of feeding can occur if any of these aspects of early feeding is impaired. Feeding problems often emerge during key feeding transitions, such as moving from breast- or bottle-feeding to cup-feeding, starting solid foods, or at the initiation of self-feeding.

It is important to distinguish clinically relevant feeding problems from developmentally typical behaviours that parents nevertheless find troublesome. Refusal of new foods (neophobia) affects many toddlers towards the end of the first year, but often resolves with time. “Picky eating” is the rejection of both familiar and unfamiliar foods, leading to inadequate variety or quantity of foods consumed, which too is common among infants and toddlers (29, 30).

In contrast, severe and prolonged feeding disturbances are classified as either “avoidant/restrictive food intake disorder” (ARFID) (29) or pediatric feeding disorder (PFD) (31). ARFID is defined as persistent failure to meet appropriate nutritional and/or energy (calorie) requirements, alongside any of: failure to achieve expected weight gain or weight faltering; significant nutritional deficiency; dependence on nutritional support (e.g., tube-feeding or nutritional supplements); or marked interference with psychosocial functioning. Feeding disturbances can occur in any of, or all of, three domains: feeding/eating too little (poor appetite and low interest in feeding/food); eating a very restricted number of foods (avoidance of foods based on sensory sensitivity, such as texture or taste); or fear of feeding/eating due to conditioned aversion (often following unpleasant consequences such as vomiting or abdominal pain). For ARFID to be diagnosed the feeding disturbance cannot be caused solely by a concurrent medical or developmental condition or must exceed that which would be expected with the condition; lack of available food and culturally sanctioned practice must also be ruled out. PFD is broader than ARFID and includes feeding disturbances directly caused by medical co-morbidities and developmental delay, as well as those occurring in healthy infants who are otherwise developing as expected. PFDs are defined as the inability to consume sufficient food and liquids to meet nutritional and hydration requirements (e.g., infant is growing slower than expected or experiencing weight faltering), and include feeding impairments linked to any of medical, nutritional, skill-based or psychosocial dysfunction. Chronic (vs. acute) PFDs persist for ≥ 3 months. Both ARFID and PDF are relatively new definitions (first appearing in 2013 and 2019, respectively).

As well as differences in the developmental course of the three RD types, most individuals have one or other, not all three. Their prevalence varies according to the definition and methods used, but parents typically report excessive crying in around 10%–20% of 1-3-month-old infants (26, 32), and sleep problems in about 30% of 6-12-month-old infants (28, 33, 34). About 25% of parents report a feeding difficulty of some kind in otherwise healthy infants (1), and up to 50% of infants and toddlers are reported to be “picky eaters” (30). In comparison, in a large cohort of five-month-old German infants, 4.2% had both sleep and feeding problems, 4.6% crying and sleeping problems, 3.9% crying and feeding problems, and just 1.9% had all three RDs (22).

In the long-term, the causes underlying these differences between RD cases are an important target for research. There is already evidence that infants and toddlers with multiple and persistent RDs are particularly likely to have serious long-term impairments such as attention, cognitive and behavioural problems (3, 4, 35). This alone indicates a need to prioritise identification and support for these cases. In addition, it suggests that some individuals have impaired physiological and/or psychological regulatory systems which are responsible both for their early RDs and for later impairments in their social, emotional and behavioural development. However, that does not seem to be true for most cases with single crying, sleeping or feeding RDs (4, 36–40). The peak in infant crying in the early weeks typically resolves without any intervention by 3–4 months of age, and most infants who cry a lot at this early stage then develop normally (36, 38, 41). Crying problems do occur at later ages, but are much rarer and are associated with long-term mental and behavioural impairments, suggesting that these cases may differ from those whose crying is confined to the early months (40, 42–45). Most infant and toddler sleep problems are transitory or intermittent, so that only around 5% have sleep problems across multiple ages (46–48). However, severe and persistent sleeping problems do predict later disorders, particularly anxiety and emotional disorders, in some cases (49, 50). Chronic picky eating (from infancy or toddlerhood to childhood), also affects a small number of children (approximately 3%–4%) (30, 51, 52): for most, this is a transient phase. Yet, single infant feeding RDs, too, are sometimes associated with emotional and behavioural disturbances later in childhood (53, 54). What distinguishes these cases remains uncertain, although here too RD severity may be a key factor. For example, one study found that 63% of infants diagnosed with ARFID between 1.5 and 3 years of age continued to have moderate or severe malnutrition at 11 years of age (53). Adults seeking treatment for ARFID commonly report longstanding highly selective feeding beginning in early infancy, such as only accepting one type of formula, or having difficulty transitioning from exclusive milk-feeding onto solid foods (55).

Unless this heterogeneity between, and even within, the three RDs is explicitly recognised, the risk is that infants found to have them will be thought of as a homogeneous group with a common underlying neuro-behavioural and psychological condition needing a common treatment. Indeed, some reports have already grouped individuals with single and multiple RDs together without acknowledging their diversity.

Because RDs have only recently been recognised by researchers and clinicians, we lack routine surveillance methods for identifying them. It follows that research studies have had to devote considerable resources to finding cases, particularly those with multiple problems which are much rarer. This alone makes it easy to understand why some recent studies have amalgamated multiple and persistent RD cases (22, 35). Yet, there is already evidence that single crying, sleeping and feeding problems often involve normal individual differences in developmental processes, while infant learning is more critical for sleeping RDs, and some forms of feeding disorders, than for excessive crying in early infancy (1, 17, 56, 57). It follows that any remedial intervention needs to take these differences, as well those between single and multiple RD cases, into account. Clinicians need to know that most RD cases they encounter will involve single problems, and to have resources available to identify them and to deliver a suitably tailored, cost-effective, intervention. In turn, being given this guidance by their trusted healthcare professional should provide many parents with immediate reassurance and practical help. We will revisit below what such a service might look like.

## Parental contributions to infant and child RDs

1. Identification of RDs and Impact on Healthcare Services.

For obvious reasons, parents, not infants or young children, approach healthcare services for guidance and are responsible for the associated costs. Ideally these initial contacts should lead to objective measures to distinguish the infant behaviours and identify the physiological and psychological factors underlying them. Existing methods, such as audio and video recording, polygraphy, and actigraphy can provide some evidence of this type (58–60). However, these methods are currently cumbersome, expensive, have their own limitations (61), and are not widely used. Instead, most studies have relied on parental reports of RDs, measured as yes/no responses or ratings of RD severity (e.g., none/mild; moderate; severe), overlooking the evidence that individual parental characteristics and vulnerabilities influence how parents perceive, respond to, and report infant RDs (62–64). It follows that, where a parental report is all that is available, it is unclear how far it measures an infant or parent. This is less critical for clinical purposes, since clinicians start with a parental report of a RD, then seek to unravel the infant, parental, and situational factors involved step by step. Further, interventions for RDs are almost always delivered by parents, so that supporting them is likely to be inherent in any intervention. Fortunately, methods for screening parental wellbeing are available for clinical use and can be included in the intervention process. For research trying to understand the infant part of RDs, however, objective measures which distinguish infant from parental components of RDs are needed. The implication is that parental reports are a key part of clinical practice, but should be thought of as “red flags” identifying families in need of support rather than as definitive measures of infants or children.

2. The clearest evidence that parental responses to RDs may sometimes contribute to good or poor outcomes at later ages comes from randomised controlled trials (RCTs). In particular, there is substantial RCT evidence that intervention programmes based on behavioural principles and delivered by parents are effective in improving night-time sleeping in infants 6 months old or older (27, 56). There are, however, caveats to this conclusion:
  • The findings apply in the short to medium term, but often wash out long-term, such as by 9 years of age (65). This leaves unclear whether parents did not sustain their allotted parenting methods, or whether the child's underlying vulnerabilities re-asserted themselves.
  • Although many parents find behavioural programmes helpful and effective (66–68) some find them unacceptable and consider their disadvantages to outweigh their benefits (69).
  • In infants under six months old, most RCTS have found behavioural interventions effective in preventing sleep problems (57, 70–75) but some found little or no benefit (76–78). This may be because of infant immaturity, because many parents experiment with a variety of parenting methods during early infancy (79, 80), or because these studies failed to achieve significant differences between intervention and control groups. Parents sometimes omit to follow all the recommendations involved in behavioural programmes (17), making RCTs involving infants particularly hard to carry out. When planning clinical interventions, parents’ individual and culturally-based judgements need to be taken into account.

In sum, these RCT findings indicate that behavioural methods can be recommended by professionals to help many families to manage infant sleep problems in the short to medium term (65). As well as this evidence, clinicians need to assess parents’ preferences, and discuss other ways of coping, from the outset (47).

With regard to feeding problems, the consensus among clinicians is that a parent need not worry if a child is:

• Eating something from each of the main food groups on most days (vegetables or fruit; potatoes, pasta, bread or rice; meat, fish or pulses; milk, cheese or yoghurt)
• Gaining weight as expected (i.e., following their weight centile)
• Active and healthy

However, many parents find feeding problems to be stressful and there is clinical and epidemiological evidence that parents who feel anxious about their infant's food or milk intake may adopt controlling (e.g., pressuring or force-feeding) or indulgent feeding practices (e.g., cajoling or making a different meal), which can prolong or exacerbate feeding problems (6, 9, 64). Instead, responsive feeding—responding appropriately, quickly, and sensitively to an infant's feeding cues—is considered by professional organisations worldwide to be the optimum approach for parents to follow to support infants in developing healthy feeding behaviours. With responsive feeding, parents offer age-appropriate foods, model eating, provide structure (what, when and where to feed) and set reasonable limits; infants are allowed to reject or accept foods, and to self-feed without pressure. Responsive feeding is a core recommendation for the prevention and treatment of infant feeding problems, with parents being advised to focus on *how* to feed rather than the amount of food consumed by the infant (64).

Within a responsive feeding framework, a helpful recent review has summarised the different behavioural strategies parents could implement for each type of feeding problem (64). The UK's Scientific Advisory Committee on Nutrition, too, has recommended: (1) *repeated exposure* to new foods (giving the infant many opportunities to try a new food, without pressure) is important for their acceptance; and (2) offering a *variety* of foods helps infants increase their acceptance of new flavours (81). A single RCT found that following a “baby-led weaning approach” (letting the infant self-feed from the start of complementary feeding) resulted in earlier self-feeding, less pickiness and greater enjoyment of food at 2 years of age, compared to traditional spoon-feeding (65). More RCTs are needed, but this evidence indicates that parents can be supported in preventing or resolving feeding difficulties if they arise and, at the very least, not making them worse (44).

Altogether, the evidence above makes it plausible that parenting in response to early RDs may sometimes influence later child development outcomes. It also points to parenting strategies which may form part of a clinical intervention which is helpful for many RD cases involving sleep or feeding problems in at least the short to medium term.

Twin studies are powerful designs for disentangling the relative contribution of genetic and environmental influences on variation in behaviour, and there have been several such studies of infant sleep and feeding (although not infant crying). On the whole, these studies indicate that both genetic and environmental factors are important, although their relative influence varies across the behaviours. In infancy, genetic factors explain only 17% of the variation in parent-reported sleep duration, with environmental factors playing the most important role and, in particular, those that are shared entirely by co-twins (e.g., parental sleep hygiene practices) (82). In contrast, genetic factors play a major role in shaping variation in some feeding behaviours, explaining 53%–84% of individual differences in enjoyment of feeding, responsiveness to feeding cues, satiety sensitivity and feeding speed, during the period of exclusive milk-feeding at around 3 months of age (18). Picky eating and neophobia also show moderate to high genetic influence (46% and 58% respectively) in toddlerhood (16 months), while environmental influences shared by co-twins are more important for picky eating (46%) than for neophobia (22%) (19). In the first longitudinal study of picky eating and neophobia combined, spanning 5 age points from toddlerhood to early adolescence, shared environmental influence was only observed at 16 months of age, but had disappeared by early childhood; on the other hand, genetic influences on picky eating and neophobia increased over time (e.g., from 60% at 16 months of age to 84% by 3 years of age) (20). The most recent systematic review, too, has concluded that feeding disorder causation typically involves a complex process of interplay between genetic and parenting factors (83). There is no evidence of shared environmental influence on ARFID; rather, susceptibility to this disorder is largely heritable (79%), at least in childhood (21). Collectively, these twin studies indicate that parents may find it easier to resolve sleep problems than feeding problems, and that feeding problems may be more modifiable in infancy and toddlerhood than in childhood. These studies also underline that parents are often not to blame for the onset of feeding problems, and that they may need considerable support with managing them.

Although RCTs and behaviour genetic studies provide strong evidence about causation, they are difficult and costly to implement, particularly where long-term follow-up is involved. Instead, most studies, particularly of multiple RDs, have employed observational designs, often prospectively at successive ages, using statistical analyses to unravel the contribution of infant and parental factors. In particular, the Bavarian Longitudinal Study has followed up a large cohort of medically at-risk infants and families from birth (35, 84), incorporating normative community groups and a parallel study in Finland for comparison. Strengths include some use of objective measures and reports from professionals to substantiate parental reports. In their most recent publication, this study has followed up the participating infants to 26–30 years of age (22). Broadly, these researchers propose a “cascade” model whereby early multiple and persistent RDs predispose some individuals to emotional, behavioural, and attentional problems in childhood, which then predispose them to psychological, behavioural and social regulatory disorders as adolescents and adults, independent of the contribution of parenting factors. These conclusions are reminiscent of the New York Longitudinal Study of temperament in the 1960s-80s, which first provided scientific credence for the view among parents that some children are constitutionally difficult. However, with the advances in methodology and conceptual modelling employed by the Bavarian study, their conclusions are particularly authoritative.

While the Bavarian study may be the leading one of its kind, a 2020 systematic review of relationships between parenting behaviour and infant regulation examined 107 studies, most of which were observational (5). They found few consistent results. They were unable to draw any conclusions about the contribution of inadequate parenting to infant RD development, but found evidence of a relationship between positive parenting behaviour and positive infant self-regulation, with differences according to age, measurement method and infant behaviour. Specifically, maternal sensitivity, responsiveness, supportiveness and positive affect were associated with good infant self-regulation. Whether this relationship reflects effects of parenting on infant behaviours, indicates that well-regulated infants are easier for parents to care for, or is due to a third factor such as shared genetic influence, remains unclear. Rather, the implication of this finding is to point to the need for more methodologically robust studies of how RDs affect parental emotions and coping behaviours, as well as studies of positive and negative parenting.

## Targeting healthcare interventions for RDs

Arising from the points made above, an obvious question is whether infant crying, sleeping and feeding problems are problems for infants or parents? The starting point, at least for healthcare interventions, is to anticipate “both”. Despite calling them infant problems, the immediate impact of RDs is on parents. Although many parents cope, there is evidence that substantial numbers will experience distress, frustration, anxiety and/or depression (12, 13, 85), while a small minority will harm the infants in their care (86). Even if the infants involved are healthy, parental wellbeing is in their best interests. Regardless, too, of whether parents maintain infant RDs, they have to cope with them on a daily basis and, in most cases, to deliver the intervention programme clinicians recommend. The lack of evidence that parenting causes infant RDs needs to be kept in mind, since parents who perceive clinicians to be blaming them, or who lose trust in their clinician, are unlikely to maintain involvement as partners in supporting their infant's development.

Historically, recognition of the need to focus on parents as part of RDs can be traced back to the need to prevent “Shaken Baby Syndrome”, now known as Abusive Head Trauma (87). This form of infant abuse was found to result in brain injury and often triggered by infant crying (88). Studies of ways to raise parental awareness of the dangers of Abusive Head Trauma, and evaluations of their effectiveness, followed (88). Other adverse outcomes of infant crying reported include abandoning breast feeding, over-feeding, and impaired parent-child relationships and child development (89). Since that time, the list of RDs has broadened to include other behaviours which challenge parents, and services to safeguard infants and children have been introduced by many healthcare providers (90).

The focus on parents with infants requires traditional professional boundaries to be crossed, to join up paediatric and adult mental health services. As these joined-up services embed in healthcare systems, there is a need to consider what form they should take and who should provide them. Their nature is likely to depend on the healthcare systems involved and the resources available to them. Because we are most familiar with the UK National Health Service, we will focus on the experience and evidence accumulated in the UK, but the core requirements seem likely to be similar across countries and healthcare systems.

Requirement 1: The services need to be embedded in primary healthcare, that is, in provisions which provide front-line surveillance, monitoring and safeguarding for all parents with infants and young children, with links to tertiary services where specialised treatments are needed. In the UK, General Practitioner doctors, community nurse Midwives and Health Visitors, and allied professionals, provide services of this sort. In particular, Health Visitors provide statutory healthcare for all families at ages which coincide with infant RDs, traditionally involving home visits which allow them an opportunity for surveillance. Approached from an adult mental health perspective, the UK Maternal Mental Health Alliance, and Managed Clinical Network for Perinatal Mental Health in Scotland, have developed services to support parental (particularly maternal) mental health in the community (91–93). These adult services do not at present include an explicit focus on infant RDs, in spite of evidence that irritable, unsettled infants can trigger mental health impairments in vulnerable mothers (94). They do not yet provide joined-up services, but have the potential to develop them.

Requirement 2: The services must be shown to be effective, and cost-effective, in supporting families tackling RDs. In particular, where attention to RDs needs to be added to existing workloads, the service needs to deliver better value for money. As an example, studies of interventions to prevent Abusive Head Trauma in Canada and North America have published evidence that the interventions are cost-effective over the longer-term (95). In the UK, the National Institute for Health and Care Excellence recommends a stepped-care model for parental wellbeing support, including during the postnatal period (92). This model involves use of low-cost supports in most cases, with more expensive interventions, such as direct one-to-one contacts, reserved for cases with additional needs. Fortunately, suitable online healthcare provisions, such as websites and apps, have coincided with these recommendations (96–99).

Requirement 3: the service needs to include two main elements: (i) gathering information about the infant behaviours involved, including their impact on parents and the need for further steps to support infant wellbeing; (ii) exploring and supporting parents’ coping, including their emotions, thoughts and actions. Many parents find RDs stressful and they are associated with parental frustration and poor mental health (12, 13, 85). While the direction of causation remains uncertain, interventions need to assess, support and safeguard both infants and parents.

Clinical groups in America and Germany have developed family interventions designed to support both parents and infants and children, including those with RDs (100, 101). Because they are delivered by clinicians directly to parents, they may be relatively expensive, particularly for primary care use in the general community. To date, most of the evidence of their effectiveness has come from case-studies without control groups, but one controlled study compared three such treatments with routine services when delivered to 193 mothers with postpartum depression. The treatments included interaction guidance based on Mc Donough's (101) principles, a psychodynamic treatment which included a focus on parental attachment, a non-directive counselling approach, and treatment as usual in the UK NHS (102). Compared to routine services, all three interventions reduced maternal depression at 4.5 months, although only psychodynamic therapy met clinical criteria for improvement. However, none of the interventions continued to show improvements at the 9-month outcome assessments and none of them improved on the spontaneous rate of remission from depression at longer-term (9, 18 or 60 month) outcomes. Similarly, a randomised controlled trial of a psychological intervention to improve maternal depression in the UK found improvements, but not clinically significant long-term benefits (103). It is plausible that cases where maternal postnatal depression is triggered by an infant RD will remit if the RD involved resolves, but the evidence available does not, as yet, confirm that explanation.

## An example: the Surviving Crying (SC) service and study

Because of the fledgling nature of services to support parents of infants with RDs, this intervention is briefly described to give a blueprint for what a service for families with RDs might look like. As its title indicates, the intervention was developed to support parents with infants they judge to be crying excessively. This includes recognising that infant crying, and parental concern about it, peak in the first few postnatal months, and that interventions involving parenting can be helpful, but not reliable in stopping the “unsoothable” crying bouts which are the primary concern for many parents (85, 104). It follows that interventions for this RD type need to be delivered early, rapidly, and to target helping parents to cope in getting through this trying period.

The SC intervention includes three main elements: a website, a booklet based on the website, and a short programme of Cognitive Behaviour Therapy (CBT) sessions delivered to parents by a specially trained healthcare professional, such as a Health Visitor (89). All participating parents are provided with the website and booklet, while about half choose to have the, more expensive, CBT sessions (89). CBT-based methods were selected because of evidence of their effectiveness in supporting adult mental health and wellbeing, including parents during the postnatal period, and because they are recommended for this purpose by the National Institute for Health and Care Excellence (105).The CBT-based sessions with parents follow a manual designed to provide the professional with step-by-step guidance. Typically, sessions start with history-taking to identify the crying features worrying the parent, since these are what have brought the parent to seek help. As well as the crying amount, this commonly includes parent's inability to soothe the baby, concern that the crying is a sign of ill health, worry that the baby is not getting enough to eat, and anxiety that the crying is the parent's fault and reflects inadequate parenting. The Crying Patterns Questionnaire (25) provides a brief standardised, freely available, interview for collecting this information. This is used to assess the crying, show parents their concerns are being taken seriously, confirm the infant's health, provide reassurance and guidance, and identify any follow-up or referral needs. When used at successive ages, it allows progress to be tracked.

This first part of the session needs to be tailored to the RD in question. The Brief Infant Sleeping Questionnaire (34) is suitable for screening for infant sleep problems, and the Infant and Child Feeding Questionnaire can identify infants or children at risk of a paediatric feeding disorder (106). “What matters to me” is a questionnaire developed by parents of children with ARFID (107) to capture their concerns, perceived impact on their child and themselves, and their hopes for treatment/goals, which can be used to assess outcomes that parents value highly.

The second part of each session turns attention to the parent(s), using CBT-based techniques to support them in developing coping strategies which help them manage their emotions and actions and ensure their own and their infant's wellbeing. Steps to take if the baby's crying becomes overwhelming are prominent both in the CBT-based sessions and website/booklet. Standardised questionnaire assessments of depression and anxiety are used to track parents’ mental health and wellbeing. This part of the intervention appears likely to be broadly similar across different RD types.

The findings from this study so far are that receipt of the SC materials was associated with improvements in parental mental health and coping, that both parents and Health Visitors wanted the SC materials to be included in the NHS, and that Health Visitors could be trained, mostly online, to deliver the materials successfully under routine NHS conditions (108). Supported by the UK National Institute for Health and Care Research, a randomised controlled trial to evaluate the effectiveness and cost of the resulting SC service is underway. Further information is available on request.

## References

[1] Kerzner B Milano K MacLean WC Berall G Stuart S Chatoor I. A practical approach to classifying and managing feeding difficulties. Pediatrics. (2015) 135(2):344–52. 10.1542/peds.2014-1630 25560449

[2] Morris S St James-Roberts I Sleep J Gillham P. Economic evaluation of strategies for managing crying and sleeping problems. Arch Dis Child. (2001) 84:15–9. 10.1136/adc.84.1.15 11124777 PMC1718606

[3] Galling B Brauer H Struck P Krogmann A Gross-Hemmi M Prehn-Kristensen A The impact of crying, sleeping, and eating problems in infants on childhood behavioral outcomes: a meta-analysis. Front Child Adolesc Psychiat. (2023) 1:1099406. 10.3389/frcha.2022.1099406 PMC11732157 39817282

[4] Hemmi MH Wolke D Schneider S. Associations between problems with crying, sleeping and/or feeding in infancy and long-term behavioural outcomes in childhood: a meta-analysis. Arch Dis Child. (2011) 96:622–9. 10.1136/adc.2010 21508059

[5] Samdan G Kiel N Petermann F Rothenfußer S Zierul C Reinelt T. The relationship between parental behavior and infant regulation: a systematic review. Dev Rev. (2020) 57:10093. 10.1016/j.dr.2020.100923

[6] Costa A Oliveira A. Parental feeding practices and children’s eating behaviours: an overview of their complex relationship. Healthcare. (2023) 11(3):400. 10.3390/healthcare11030400 36766975 PMC9914567

[7] Hiscock H Wake M. Infant sleep problems and postnatal depression: a community-based study. Pediatrics. (2001) 107(6):1317–22. 10.1542/peds.107.6.1317 11389250

[8] Georg AK Schröder-Pfeifer P Cierpka M Taubner S. Maternal parenting stress in the face of early regulatory disorders in infancy: a machine learning approach to identify what matters most. Front Psychiatry. (2021) 12:663285. 10.3389/fpsyt.2021.663285 34408674 PMC8365191

[9] Kininmonth AR Herle M Tommerup K Haycraft E Farrow C Croker H Parental feeding practices as a response to child appetitive traits in toddlerhood and early childhood: a discordant twin analysis of the gemini cohort. Int J Behav Nutrit Phys Act. (2023) 20(1):39. 10.1186/s12966-023-01440-2 PMC10074660 37016417

[10] Martini J Petzoldt J Knappe S Garthus-Niegel S Asselmann E Wittchen HU. Infant, maternal, and familial predictors and correlates of regulatory problems in early infancy: the differential role of infant temperament and maternal anxiety and depression. Early Hum Dev. (2017) 115:23–31. 10.1016/j.earlhumdev.2017.08.005 28869923

[11] Olsen AL Ammitzbøll J Olsen EM Skovgaard AM. Problems of feeding, sleeping and excessive crying in infancy: a general population study. Arch Dis Child. (2019) 104(11):1034–41. 10.1136/archdischild-2019-316851 31270094

[12] Petzoldt J Wittchen HU Einsle F Martini J. Maternal anxiety versus depressive disorders: specific relations to infants’ crying, feeding and sleeping problems. Child Care Health Dev. (2016) 42(2):231–45. 10.1111/cch.12292 26490836

[13] Powell C Bamber D Long J Garratt R Brown J Rudge S Mental health and well-being in parents of excessively crying infants: prospective evaluation of a support package. Child Care Health Dev. (2018) 44(4):607–15. 10.1111/cch.12566 29667223

[14] Stein A Woolley H Murray L Cooper P Cooper S Noble F Influence of psychiatric disorder on the controlling behaviour of mothers with 1-year-old infants: a study of women with maternal eating disorder, postnatal depression and a healthy comparison group. Br J Psychiatry. (2001) 179:157–62. 10.1192/bjp.179.2.157 11483478

[15] Korja R Nolvi S Grant KA McMahon C. The relations between maternal prenatal anxiety or stress and child’s early negative reactivity or self-regulation: a systematic review. Child Psychiatry Hum Dev. (2017) 48(6):852–9. 10.1007/s10578-017-0709-0 28124273

[16] St James-Roberts I Conroy S Wilsher K. Links between maternal care and persistent infant crying in the early months. Child Care Health Dev. (1998) 24(5):353–76. 10.1046/j.1365-2214.2002.00089.x 9728283

[17] St. James-Roberts I Alvarez M Csipke E Abramsky T Goodwin J Sorgenfrei E. Infant crying and sleeping in London, Copenhagen and when parents adopt a ‘proximal’ form of care. Pediatrics. (2006) 117(6):e1146–55. 10.1542/peds.2005-2387 16740816

[18] Llewellyn CH Van Jaarsveld CHM Johnson L Carnell S Wardle J. Nature and nurture in infant appetite: analysis of the gemini twin birth cohort. Am J Clinl Nutrition. (2010) 91(5):1172–9. 10.3945/ajcn.2009.28868 20335548

[19] Smith AD Herle M Fildes A Cooke L Steinsbekk S Llewellyn CH. Food fussiness and food neophobia share a common etiology in early childhood. J Child Psychol Psychiatry. (2017) 58(2):189–96. 10.1111/jcpp.12647 27739065 PMC5298015

[20] Nas Z Herle M Kininmonth A Smith A Bryant-Waugh R Fildes A Nature and nurture in fussy eating from toddlerhood to early adolescence: findings from the gemini twin cohort. PsyArXiv. (2023) 1:1–36. 10.31234/osf.io/ac7vy PMC11754699 39299707

[21] Dinkler L Wronski ML Lichtenstein P Lundström S Larsson H Micali N Etiology of the broad avoidant restrictive food intake disorder phenotype in Swedish twins aged 6 to 12 years. JAMA Psychiatry. (2023) 80(3):260–9. 10.1001/jamapsychiatry.2022.4612 36723946 PMC9978946

[22] Wolke D Baumann N Jaekel J Pyhälä R Heinonen K Räikkönen K The association of early regulatory problems with behavioral problems and cognitive functioning in adulthood: two cohorts in two countries. J Child Psychol Psychiatry. (2023) 64(6):876–85. 10.1111/jcpp.13742 36601777

[23] Brazelton TB. Crying in infancy. Pediatrics. (1962) 29(4):579–88. 10.1542/peds.29.4.579 13872677

[24] Barr RG. The normal crying curve: what do we really know? Dev Med Child Neurol. (1990) 32:356–62. 10.1111/j.1469-8749.1990.tb16949.x 2332126

[25] St James-Roberts I Halil T. Infant crying patterns in the first year: normal community and clinical findings. J Child Psychol Psychiat. (1991) 32(6):951–68. 10.1111/j.1469-7610.1991 1744198

[26] Wolke D Bilgin A Samara M. Systematic review and meta-analysis: fussing and crying durations and prevalence of colic in infants. J Ped. (2017) 85:55–61e4. 10.1016/j.jpeds.2017.02.020 28385295

[27] Sadeh A. Maturation of normal sleep patterns in childhood through adolescence. In: Loughlin G Carroll J Marcus CL, editors. Sleep & Breathing in Children: A Developmental Approach. New York: Marcel Dekker (2000). p. 2–25.

[28] Zuckerman B Stevenson J Bailey V. Sleep problems in early childhood: continuities, predictive factors, and behavioral correlates. Peds. (1987) 80(5):664–71. 10.1542/peds.80.5.664 3670967

[29] American Psychiatric Association. Diagnostic and Statistical Manual of Mental Disorders. 5th edn Washington, DC: American Psychiatric Association (2013).

[30] Taylor CM Wernimont SM Northstone K Emmett PM. Picky/fussy eating in children: review of definitions, assessment, prevalence and dietary intakes. Appetite. (2015) 95:349–59. 10.1016/j.appet.2015.07.026 26232139

[31] World Health Organisation. International Statistical Classification of Diseases and Related Health Problems. Geneva: World Health Association (2019).

[32] Douglas P Hill P. Managing infants who cry excessively in the first few months of life. Br Med J. (2011) 343:d7772–d7772. 10.1136/bmj.d7772 22174332

[33] Armstrong KL Quinn RA Dadds MR. The sleep patterns of normal children. Med J Australia. (1994) 161(3):202–6. 10.5694/j.1326-5377.1994.tb127383.x 8035724

[34] Sadeh A. A brief screening questionnaire for infant sleep problems: validation and findings for an internet sample. Peds. (2004) 113:e570. 10.1542/peds.113.6.e570 15173539

[35] Schmid G Schreier A Meyer R Wolke D. Predictors of crying, feeding and sleeping problems: a prospective study. Child Care Health Dev. (2011) 37(4):493–502. 10.1111/j.1365-2214.2010.01201.x 21299592

[36] Bell G Hiscock H Tobin S Cook F Sung V. Behavioral outcomes of infant colic in toddlerhood: a longitudinal study. J Peds. (2018) 201:154–9. 10.1016/j.jpeds.2018.05.010 29887386

[37] Elliott MR Pedersen EL Mogan J. Early infant crying: child and family follow-up at three years. Canad J Nurs Res. (1997) 29(2):47–67. 9355290

[38] Lehtonen L. Clinical pies for etiology and outcome in infants presenting with early increased crying. In: Barr R Hopkins B Green J, editors. Crying as a Sign, a Symptom, and a Signal. London: MacKeith Press/Cambridge University Press (2000). p. 67–95.

[39] Scher A Zukerman S Epstein R. Persistent night waking and settling difficulties across the first year: early precursors of later behavioural problems? J Reprod Infant Psychol. (2005) 23(1):77–88. 10.1080/02646830512331330929

[40] Von Kries R Kalies H Papoušek M. Excessive crying beyond 3 months may herald other features of multiple regulatory problems. Arch Pediatr Adolesc Med. (2006) 160(5):508. 10.1001/archpedi.160.5.508 16651494

[41] St James-Roberts I Peachey E. Distinguishing infant prolonged crying from sleep-waking problems. Arch Dis Child. (2011) 96(4):340–4. 10.1136/adc.2010.200204 21220260 PMC3202670

[42] Rao MR Brenner RA Schisterman EF Vik T Mills JL. Long term cognitive development in children with prolonged crying. Arch Dis Child. (2004) 89(11):989–92. 10.1136/adc.2003.039198 15499048 PMC1719720

[43] Santos IS Matijasevich A Capilheira MF Anselmi L Barros FC. Excessive crying at 3 months of age and behavioural problems at 4 years age: a prospective cohort study. J Epidemiol Community Health. (2015) 69:654–9. 10.1136/jech-2014-204568 25700531 PMC4484259

[44] Smarius LJCA Strieder TGA Loomans EM Doreleijers TAH Vrijkotte TGM Gemke RJ Excessive infant crying doubles the risk of mood and behavioral problems at age 5: evidence for mediation by maternal characteristics. Eur Child Adolesc Psychiatry. (2017) 26(3):293–302. 10.1007/s00787-016-0888-4 27422707 PMC5323467

[45] Wolke D Rizzo P Woods S. Persistent infant crying and hyperactivity problems in middle childhood. Peds. (2002) 109(6):1054–60. 10.1542/peds.109.6.1054 12042542

[46] Jenkins S Owen C Bax M Hart H. Continuities of common behaviour problems in preschool children. J Child Psychol Psychiat. (1984) 25(1):75–90. 10.1111/j.1469-7610.1984.tb01720.x 6607263

[47] St James-Roberts I. The Origins, Prevention and Treatment of Infant Crying and Sleeping Problems. New York, NY: Routledge/Taylor & Francis Group (2012).

[48] Wake M Morton-Allen E Poulakis Z Hiscock H Gallagher S Oberklaid F. Prevalence, stability, and outcomes of cry-fuss and sleep problems in the first 2 years of life: prospective community-based study. Peds. (2006) 117:836–42. 10.1542/peds.2005-0775 16510665

[49] Cook F Conway LJ Giallo R Gartland D Sciberras E Brown S. Infant sleep and child mental health: a longitudinal investigation. Arch Dis Child. (2020) 105(7):655–60. 10.1136/archdischild-2019-318014 32152038

[50] Sidor A Fischer C Cierpka M. The link between infant regulatory problems, temperament traits, maternal depressive symptoms and children’s psychopathological symptoms at age three: a longitudinal study in a German at-risk sample. Child Adolesc Psychiatry Ment Health. (2017) 11:10. 10.1186/s13034-017-0148-5 28286548 PMC5340036

[51] Bourne L Bryant-Waugh R Mandy W Solmi F. Investigating the prevalence and risk factors of picky eating in a birth cohort study. Eat Behav. (2023) 50:101780. 10.1016/j.eatbeh.2023.101780 37453176

[52] Cano S C Tiemeier H Van Hoeken D Tharner A Jaddoe VWV Hofman A Trajectories of picky eating during childhood: a general population study. Int J Eat Disords. (2015) 48(6):570–9. 10.1002/eat.22384 25644130

[53] Lucarelli L Sechi C Cimino S Chatoor I. Avoidant/restrictive food intake disorder: a longitudinal study of malnutrition and psychopathological risk factors from 2 to 11 years of age. Front Psychol. (2018) 9:1608. 10.3389/fpsyg.2018.01608 30233458 PMC6127741

[54] Ammaniti M Lucarelli L Cimino S D’Olimpio F Chatoor I. Feeding disorders of infancy: a longitudinal study to middle childhood. Int J Eat Disords. (2012) 45(2):272–80. 10.1002/eat.20925 21495054

[55] Thomas JJ Lawson EA Micali N Misra M Deckersbach T Eddy KT. Avoidant/restrictive food intake disorder: a three-dimensional model of neurobiology with implications for etiology and treatment. Curr Psychiatry Reps. (2017) 19:1–9. 10.1007/s11920-017-0753-2 PMC6281436 28714048

[56] Mindell JA Kuhn B Lewin DS Meltzer LJ Sadeh A. Behavioral treatment of bedtime problems and night wakings in infants and young children. Sleep. (2006) 29:1263–76. 17068979

[57] St. James-Roberts I Sleep J Morris S Owen C Gillham P. Use of a behavioural programme in the first 3 months to prevent infant crying and sleeping problems. J Paediatr Child Health. (2001) 37(3):289–97. 10.1046/j.1440-1754.2001.00699.x 11468047

[58] Anders TF Halpern LF Hua J. Sleeping through the night: a developmental perspective. Peds. (1992) 90(4):554–60. 10.1542/peds.90.4.554 1408509

[59] Kirjavainen J Kirjavainen T Huhtala V Lehtonen L Korvenranta H Kero P. Infants with colic have a normal sleep structure at 2 and 7 months of age. J Peds. (2001) 138(2):218–23. 10.1067/mpd.2001.110326 11174619

[60] St James-Roberts I Conroy S Wilsher K. Clinical, developmental and social aspects of infant crying and colic. Early Dev Parent. (1995) 4(4):177–89. 10.1002/edp.2430040404

[61] de Souza L Benedito-Silva AA Pires MLN Poyares D Tufik S Calil HM. Further validation of actigraphy for sleep studies. Sleep. (2003) 26:81–5. 10.1093/sleep/26.1.81 12627737

[62] Pauli-Pott U Becker K Mertesacker T Beckmann D. Infants with ‘colic’ - mothers’ perspectives on the crying problem. J Psychosom Res. (2000) 48(2):125–32. 10.1016/S0022-3999(99)00084-7 10719128

[63] Zwart P Vellema-Goud MGA Brand PLP. Characteristics of infants admitted to hospital for persistent colic, and comparison with healthy infants. Acta Paediatr. (2007) 96(3):401–5. 10.1111/j.1651-2227.2007.00090.x 17407465

[64] Milano K Chatoor I Kerzner B. A functional approach to feeding difficulties in children. Curr Gastroenterol Reps. (2019) 21:1–8. 10.1007/s11894-019-0669-6 31444689

[65] Price AMH Wake M Ukoumunne OC Hiscock H. Five-year follow-up of harms and benefits of behavioral infant sleep intervention: randomized trial. Peds. (2012) 130(4):643–51. 10.1542/peds.2011-3467 22966034

[66] Honaker SM Schwichtenberg AJ Kreps TA Mindell JA. Real-world implementation of infant behavioral sleep interventions: results of a parental survey. J Peds. (2018) 199:106–111e2. 10.1016/j.jpeds.2018.04.009 29753539

[67] Kahn M Barnett N Gradisar M. Implementation of behavioral interventions for infant sleep problems in real-world settings. J Peds. (2023) 255:137–146.e2. 10.1016/j.jpeds.2022.10.038 36375604

[68] Stevens J Splaingard D Webster-Cheng S Rausch J Splaingard M. A randomized trial of a self-administered parenting intervention for infant and toddler insomnia. Clin Pediats (Phila). (2019) 58(6):633–40. 10.1177/0009922819832030 30782008

[69] Ball HL Taylor CE Thomas V Douglas PS. Development and evaluation of ‘sleep, baby & you’-an approach to supporting parental well-being and responsive infant caregiving. PLoS One. (2020) 15:e0237240. 10.1371/journal.pone.0237240 32764810 PMC7413483

[70] Adair R Zuckerman B Bauchner H Philipp B Levenson S. Reducing night waking in infancy: a primary care intervention. Pediatrics. (1992) 89(4):585–8. 10.1542/peds.89.4.585 1557234

[71] Pinilla T Birch LL. Help me make it through the night: behavioral entrainment of breast-fed infants’ sleep patterns. Peds. (1993) 91(2):436–44. 10.1542/peds.91.2.436 8424024

[72] Kerr SM Jowett SA Smith LN. Preventing sleep problems in infants: a randomized controlled trial. J Adv Nurs. (1996) 24(5):938–42. 10.1111/j.1365-2648.1996.tb02929.x 8933253

[73] Stremler R Hodnett E Lee K MacMillan S Mill C Ongcangco L A behavioral-educational intervention to promote maternal and infant sleep: a pilot randomized, controlled trial. Sleep. (2006) 29(12):1609–15. 10.1093/sleep/29.12.1609 17252892

[74] Symon BG Marley JE Martin AJ Norman ER. Effect of a consultation teaching behaviour modification on sleep performance in infants: a randomised controlled trial. Med J Australia. (2005) 182(5):215–8. 10.5694/j.1326-5377.2005.tb06669.x 15748130

[75] Wolfson A Lacks P Futterman A. Effects of parent training on infant sleeping patterns, parents’ stress, and perceived parental competence. J Consult Clin Psychol. (1992) 60(1):41–8. 10.1037/0022-006X.60.1.41 1556284

[76] Galland BC Sayers RM Cameron SL Gray AR Heath ALM Lawrence JA Anticipatory guidance to prevent infant sleep problems within a randomised controlled trial: infant, maternal and partner outcomes at 6 months of age. BMJ Open. (2017) 7(5):e014908. 10.1136/bmjopen-2016-014908 28576897 PMC5623410

[77] Hiscock H Cook F Bayer J Le HND Mensah F Cann W Preventing early infant sleep and crying problems and postnatal depression: a randomized trial. Peds. (2014) 133(2):e346–54. 10.1542/peds.2013-1886 24394682

[78] Stremler R Hodnett E Kenton L Lee K Weiss S Weston J Effect of behavioural-educational intervention on sleep for primiparous women and their infants in early postpartum: multisite randomised controlled trial. Br Med J. (2013) 346:f1164. 10.1136/bmj.f1164 23516146 PMC3603553

[79] Goodlin-Jones BL Burnham MM Gaylor EE Anders TF. Night waking, sleep-wake organization, and self-soothing in the first year of life. J Dev Behav Peds. (2001) 22(4):226–32. 10.1097/00004703-200108000-00003 PMC1201414 11530895

[80] St James-Roberts I Roberts M Hovish K Owen C. Video evidence that parenting methods predict which infants develop long night-time sleep periods by three months of age. Prim Health Care Res Dev. (2017) 18(3):1–15. 10.1017/S1463423616000451 28029090 PMC5966725

[81] Scientific Advisory Committee on Nutrition. Feeding Young Children Aged 1 to 5 Years. Scientific Advisory Committee on Nutrition (SACN) (2023). Available online at: Available at: https://assets.publishing.service.gov.uk/government/uploads/system/uploads/attachment_data/file/1167077/SACN-Feeding-young-children-aged-1-to-5-full-report.pdf (Access June 5, 2023). 10.1017/S0029665126102250 41748544

[82] Kocevska D Barclay NL Bramer WM Gehrman PR Van Someren EJW. Heritability of sleep duration and quality: a systematic review and meta-analysis. Sleep Med Revs. (2021) 59:101448. 10.1016/j.smrv.2021.101448 33636423

[83] Martini MG Barona-Martinez M Micali N. Eating disorders mothers and their children: a systematic review of the literature. Arch Women’s Mental Health. (2020) 23:449–67. 10.1007/s00737-020-01019-x PMC7368867 31938867

[84] Wolke D Meyer R Ohrt B Riegel K. Co-morbidity of crying and feeding problems with sleeping problems in infancy: concurrent and predictive associations. Early Dev Parent. (1995) 4(4):191–208. 10.1002/edp.2430040405

[85] Fujiwara T Barr RG Brant R Barr M. Infant distress at five weeks of age and caregiver frustration. J Peds. (2011) 159(3):425–430e2. 10.1016/j.jpeds.2011.02.010 21429518

[86] Barr RG Trent RB Cross J. Age-related incidence curve of hospitalized shaken baby syndrome cases: convergent evidence for crying as a trigger to shaking. Child Abuse Negl. (2006) 30(1):7–16. 10.1016/j.chiabu.2005.06.009 16406023

[87] Altman RL Canter J Patrick PA Daley N Butt NK Brand DA. Parent education by maternity nurses and prevention of abusive head trauma. Peds. (2011) 128(5):e1164–72. 10.1542/peds.2010-3260 22025587

[88] Barr RG Barr M Rajabali F Humphreys C Pike I Brant R Eight-year outcome of implementation of abusive head trauma prevention. Child Abuse Negl. (2018) 84:106–14. 10.1016/j.chiabu.2018.07.004 30077049

[89] St James-Roberts I Garratt R Powell C Bamber D Long J Brown J A support package for parents of excessively crying infants: development and feasibility study. Health Technol Assess. (2019) 23:56. 10.3310/hta23560 PMC6801366 31597591

[90] Lincolnshire County Council. Children’s Health Operational Management and Safeguarding Supervision Policy. Lincolnshire: Lincolnshire Children Safeguarding Partnership (2018) Available online at: Available at: https://www.lincolnshire.gov.uk/safeguarding/lscp

[91] Giridhar R MacDonagh E. Perinatal mental health services in the United Kingdom: we have only just begun. J Psychiatry Spect. (2023) 2(1):4–6. 10.4103/jopsys.jopsys_39_22

[92] National Institute for Health and Care Excellence. Antenatal and Postnatal Mental Health: Clinical Management and Service Guidance CG192. London: National Institute for Health and Care Excellence (NICE) (2014) Available online at: Available at: https://www.nice.org.uk/guidance/cg192 31990493

[93] Scott J Mcdonald C McRobbie S Watt B Young J Morris J. Stakeholder views on the design of national health service perinatal mental health services: 360-degree survey. BJPsych Bull. (2023) 48:1–6. 10.1192/bjb.2023.26 PMC10801362 37203461

[94] Murray L Cooper P. The impact of irritable infant behavior on maternal mental state: a longitudinal study and a treatment trial. In: Barr RG St James-Roberts I Keefe MR, editors. New Evidence on Unexplained Early Infant Crying: its Origins, Nature and Management. Skillman, New Jersey: Johnson & Johnson Pediatric Institute (2001). p. 149–64.

[95] Beaulieu E Rajabali F Zheng A Pike I. The lifetime costs of pediatric abusive head trauma and a cost-effectiveness analysis of the period of purple crying program in British Columbia, Canada. Child Abuse Negl. (2019) 74:104933. 10.1016/j.chiabu.2019.104133 31473380

[96] Cry-sis. *Cry-sis*. Available online at: https://www.cry-sis.org.uk (Accessed Septemeber 21, 2023).

[97] National Center on Shaken Baby Syndrome. *What is the Period of Purple Crying Programe?* Available online at: https://dontshake.org/purple-crying (Accessed Septemeber 21, 2023).

[98] raisingchildren.net.au. Available onlibe at: Available at: https://raisingchildren.net.au/ (Accessed Septemeber 21, 2023).

[99] Augustin M Licata-Dandel M Breeman LD Harrer M Bilgin A Wolke D Effects of a Mobile-based intervention for parents of children with crying, sleeping, and feeding problems: randomized controlled trial. JMIR Mhealth Uhealth. (2023) 11:e41804. 10.2196/41804 36897641 PMC10039405

[100] McDonough SC. Interaction guidance. In: Sameroff AJ McDonough SC Rosenblum KC, editors. Treating Parent-Infant Relationship Problems. New York: Guilford Press (2004). p. 79–96.

[101] Cierpka M. Treatment approaches for regulatory disorders. In: Cierpka M, editors. Regulatory Disorders in Infants. Switzerland: Springer (2016). p. 181–200. 10.1007/978-3-319-43556-5_9

[102] Cooper PJ Murray L Wilson A Romaniuk H. Controlled trial of the short and longer-term effect of psychological treatment for postpartum depression. B J Psychiat. (2003) 182:412–9. 10.1192/bjp.182.5.412 12724244

[103] Morrell C Warner R Slade P Dixon S Walters S Paley G Psychological interventions for postnatal depression: cluster randomised trial and economic evaluation. The PoNDER trial. Health Technol Assess. (2009) 13(30):1–153. 10.3310/hta13300 19555590

[104] St James-Roberts I Conroy S Wilsher K. Bases for maternal perceptions of infant crying and colic behaviour. Arch Dis Child. (1996) 75(5):375–84. 10.1136/adc.75.5.375 8957949 PMC1511785

[105] National Institute for Health and Care Excellence. Common Mental Health Problems: Identification and Pathways to Care CG123. London: National Institute for Health and Care Excellence (2011). Available online at: https://www.nice.org.uk/guidance/cg123

[106] Silverman AH Berlin KS Linn C Pederson J Schiedermayer B Barkmeier-Kraemer J. Psychometric properties of the infant and child feeding questionnaire. J Peds. (2020) 223:81–6. 10.1016/j.jpeds.2020.04.040 32507621

[107] Bryant-Waugh R Cooke L Harris M Mora M Withers F. 109 What matters to me?: development and use of a person centred outcome measure (pcom) for parents of children with feeding disorders. Arch Dis Child. (2017) 102(Suppl 3):A30–1. 10.1136/archdischild-2017-084620.81

[108] St James-Roberts I Griffiths S Watson M White C Brown J. A CBT-based training module for UK health visitors who support parents with excessively crying babies: development and initial evaluation. Prim Healthcare Res Dev. (2024). In Press.

